# Austere and Burnt Offerings: Countering the Material and Symbolic Othering Within School Meals in Aotearoa New Zealand with a Community Psychology Lens

**DOI:** 10.3390/ijerph23081011

**Published:** 2026-08-02

**Authors:** Kimberly Mary Jackson

**Affiliations:** School of Psychology, The University of Waikato, Gate 1, Knighton Road, Hamilton 3240, New Zealand; jackson.km070@gmail.com

**Keywords:** food insecurity, school meals, community psychology, neoliberalism, settler colonial societies

## Abstract

**Highlights:**

**Public health relevance—How does this work relate to a public health issue?**

- Food insecurity and poverty adversely affect children’s well-being.
- Food provision in schools is an internationally recognised method of improving children’s nutrition and health.

**Public health significance—Why is this work of significance to public health?**

- Not all school meal programmes are equally effective interventions.
- This study demonstrates how societal narratives influence policy on food in schools.
- A social justice, community psychology lens can help to understand narratives informing public health policy.

**Public health implications—What are the key implications or messages for practitioners, policymakers, and/or researchers in public health?**

- A carefully planned school meal programme has the potential to support children to flourish when the programme is culturally grounded and builds community.
- Historical, societal, and ideological narratives have influenced the design, delivery, and quality of school meals in Aotearoa New Zealand, for better and for worse.

**Abstract:**

Globally, school meals have formed a social safety net for improving children’s nutrition, health, and educational attainment. In Aotearoa New Zealand, government funded lunches for children attending schools in low-income neighbourhoods were introduced in 2020, amidst rising poverty levels and consequent household food insecurity. The aspirations encapsulated within the scheme’s indigenous Māori name, ‘Ka Ora, Ka Ako’ (‘to be well, healthy and safe; to be ready to learn’) have lost focus since reforms to the lunches in 2024 prioritised cost savings, resulting in widely publicised quality issues. This present study draws from a critical multi-level investigation into the issue of hungry children in New Zealand schools, prior to the introduction of ‘Ka Ora, Ka Ako’. Narrative analysis identified how food in schools has been conceptualised within the context of a settler colonial charity model and neoliberal austerity. Applying a critical community psychology lens highlights how burnt and meagre lunches offered to children from food insecure households are a concrete product of historically grounded, unequal relations between groups. A community psychology approach reimagines state-funded food in schools as a public good, arguing for equitable rights to nutrition, including recognition of Māori rights to food sovereignty.

## 1. Introduction

The focus of this qualitative study is the establishment and subsequent reform of a state-funded school meal programme in Aotearoa New Zealand. Community psychology provides a framework for a critical analysis that reflects a social justice orientation [1]. In addition, the present study is informed by social practice theory [2], which illuminates how macro-level processes (structural and ideological contexts) can take form within people’s everyday practices. The object of a school meal is conceptualised as a material product of broader processes, which can be examined through analysis of policy and media antecedents for the meals’ inception. The following background section introduces some relevant historical and cultural context for the state-funded meal programme, illustrating how the local experience is also situated within broader ideological developments.

### 1.1. Background

Internationally, meals in schools have been one means for ensuring children are sufficiently nourished during the school day [3]. The majority of OECD countries have national school meal programmes, although these vary from universally provided free meals to targeted funding, or subsidies for children from low-income households [4] (p. 27). Regardless of how national school meal programmes are organised, their goals are largely to improve educational equity and overall nutritional and health status, particularly for children who are disadvantaged and at risk of inadequate food within their homes [3,5].

Evidence supports positive educational benefits from providing food in schools, such as improvements in school attendance, classroom behaviour, executive function and academic performance [6,7,8]. Food in schools is also associated with positive health outcomes, although the multi-factorial nature of health determinants and variations in nutritional content make decisive causal relationships less clear, particularly in wealthier countries [9,10,11]. Longitudinal data from Sweden provide empirical evidence of wide-ranging long-term benefits from the introduction of a nutritious school meal, including improvements in health outcomes that can be connected to improved nutritional status [12]. Positive health outcomes are most notable in contexts where school meal programmes have robust quality standards [13,14].

Variations in how school meals are provided highlight that, while programmes are often introduced to meet stated health, social, educational, and practical goals, food is not simply fuel; rather, food is embedded within diverse contexts [15]. Each nation has a different history and cultural milieu, particular ways of conceptualising and organising food practices. Consequently, school meals have emerged within their specific settings, not in isolation from the broader societies in which they occur. From this standpoint, different forms of school food provision are historically and culturally grounded processes, which in turn, shape material outcomes.

An example of how school food is contextualised by its cultural setting is found in the case of Finland. A state-funded school meal programme in Finland was instituted across all levels of education, from pre-primary to upper secondary, in 1948 [16]. The meals formed part of post-war recovery, countering malnutrition and inequalities. Consequently, all children, regardless of background, are provided with a freshly prepared, high-quality meal that meets one third of their daily nutritional requirements. The meals are enshrined in education legislation and are part of pedagogy within the school day, offering nutritional, social and cultural learning through sharing meals as a school community of children and staff [17]. School meals have developed in Finland in alignment with an overall focus on equity within their education system, where cooperation is emphasised over competition and the system seeks to balance academic achievement and overall wellbeing [18].

In contrast, the United States of America (USA) has been characterised as a more individualistic society, where personal achievement and self-sufficiency are valued [19]. School meals have developed within a context where systemic social problems have proved difficult to address through collective action. While there is Federal funding to schools to provide food to children living below the poverty line, individual school districts and schools are required to provide meals, and take different approaches to the organisation and content of school food [20]. Tight budget constraints and low federal nutritional requirements have affected the quality of school food in the USA, with disproportionate impacts on students of colour [21]. The poor-quality food that characterises a ‘free lunch’ contributes to stigma for children, based on their socio-economic status [22].

In the case of Aotearoa New Zealand, providing food in schools has not been a function of the state or education system until recently [23]. School children in Aotearoa New Zealand traditionally brought a packed lunch, often sandwiches, prepared at home. Consequently, most New Zealand schools were not built with catering kitchens or dining facilities, and the small numbers of children without food (whether having forgotten their lunch or due to families being unable to provide lunch from home) were supported informally within schools, where a loaf of bread might be kept in the staffroom for such a purpose [24]. The introduction of a state-funded school meal in some schools in 2020 consequently marked a shift in how responsibility for school lunches had historically been ascribed.

#### 1.1.1. Settler Colonialism

Aotearoa New Zealand is a Pacific country, home to indigenous Māori, but colonised from the late 1700s by Europeans, predominantly from the United Kingdom [25] (p. 32), [26]. The British Crown eventually sought sovereignty and in 1840 The Treaty of Waitangi (the Māori version: Te Tiriti O Waitangi) was signed between the British Crown and chiefs from across the country. There is a complex history and set of ongoing issues stemming from Te Tiriti O Waitangi/ The Treaty of Waitangi that are beyond the scope of this article. However, two key issues are of particular relevance. Firstly, the idea that Māori at that time *ceded sovereignty*, as the British enacted it, is not credible [25] (pp. 198–200). Secondly, following the signing of The Treaty of Waitangi, a period of rapid settlement by British people, drawn by the promise of an arcadian ‘land of plenty’, began, such that by the 1870s, non-Māori outnumbered the Māori population by 10 to 1 [27]. Historians have defined this pattern, whereby colonisation is followed by the arrival of mass migration to secure control of land and resources for the colonisers, as settler colonialism. Settler colonialism is understood as a mode of domination, “part of a collective and sovereign displacement” [28]. An important feature of settler colonialism is that it is not an event (for example, the arrival of Europeans); rather, it is a structure for the continuing appropriation of a place [29].

Settler colonial power was entrenched through appropriation of Māori land by multiple means, and the suppression of Māori cultural practices and language, privileging the colonisers’ language and social customs [30]. Farming became a key economic driver and profits from the production of high-quality foods for export have benefitted some groups more than others [31]. Consequently, Aotearoa New Zealand demonstrates patterns of economic marginalisation and health inequities for the Māori population [32]. The history of how food is understood, produced, and distributed, and how scarcity is generated and responded to, takes place in the context of the rupture in Māori ways of being by the imposition of a transplanted European project [30].

Māori food customs include the concept of Manaakitanga (hospitality, welcome, generosity), which often unfolds within communal meals [33]. Food practices emphasising collectivity are protective of the well-being of the whole group [34]. Reflecting on the development of school lunch practices in Aotearoa New Zealand, the individual lunches brought from home and suiting individual preferences, each hastily consumed by children before their outdoor playtime, can be seen as a settler colonial practice. The types of foods that become established as ‘normal’ are one consequence of the packed lunch, norms that can result in stigma for groups with non-anglo food cultures [35]. Importantly, the location of responsibility for food to consume at school within individual households means that the abundance or scarcity within a child’s home determines what they eat during the day.

#### 1.1.2. Neoliberalism

Settler colonial culture finds expression in the notion of a sturdy and self-reliant individual, forging their own success within a land of opportunity [36] (p. 59). In Aotearoa New Zealand, this mythology dovetails with neoliberal ideology [37]. Although not always clearly defined, core tenets of neoliberalism are generally economic liberalisation (deregulation of markets and promotion of global free-trade), labour deregulation (flexible work arrangements and weaker employment protections), privatisation and marketisation of services, emphasis on consumer choice, and reductions in government spending [37]. The economic and political shifts encapsulated within neoliberal policies also impact at the level of individual psychology and social organisation, because an entrepreneurial, flexible worker is required [38]. Neoliberal policies often devolve responsibility from the state and place more responsibility for managing risks onto individuals [37,38]. Neoliberal reforms were introduced in Aotearoa New Zealand from the mid-1980s [39].

The advent of neoliberal policies resulted in less secure employment and lower incomes, rising housing costs, and a more stringent welfare system, engendering sharp rises in child poverty and household food insecurity from the 1990s [40]. Food insecurity refers to people lacking access to sufficient nutritionally adequate food to support health and wellbeing [41]. In Aotearoa New Zealand, food insecurity is largely related to socio-economic hardship, since food is often the only flexible item within tight household budgets [42]. The cost of staple foods in Aotearoa also rose significantly in real terms during the 2000s, particularly dairy products, meat, and fruit and vegetables [43]. In addition to burgeoning use of foodbanks and emergency welfare grants for food, schools began to report sharp rises in the numbers of children attending without breakfast or lunch [44].

#### 1.1.3. Hungry Children in Schools

Children in schools are a visible edge of food insecurity, bringing into a shared space the realities of household scarcity. Publicity about the issue of children arriving at school without food for the day intensified from the late 1990s, as the impacts of growing household food insecurity increased [41]. School principals and teachers reported the increasing scale of the problem and expressed frustrations around why they seemed to be responsible for resolving the issue [44]. By the 2010s, a multitude of schemes for feeding children in schools were developing, including those initiated by motivated individuals, community groups, non-governmental organisations, businesses, and corporate partnerships [23]. The growth of ad hoc, ground-level food-in-schools schemes highlights the lack of coordinated state-level action, although the Ministry of Health began funding a free fruit scheme for children aged 5–12 years attending schools in the most deprived areas from 2005 [45]. The government also entered into sponsored partnerships with companies in 2012 to provide breakfasts in schools in low-income neighbourhoods. Nonetheless, when a private member’s bill, The Education (Breakfast and Lunch Programmes in Schools) Amendment Bill (colloquially known as the “Feed the Kids Bill”) proposing to provide free breakfast and lunch for all children in schools from the lowest income areas was drawn from the ballot, it was not passed [46].

#### 1.1.4. Introduction of State-Funded School Meals: Ka Ora, Ka Ako

In 2020, the government expanded a 2019 school meal pilot to provide free and healthy lunches to children attending some primary schools with high levels of socio-economic disadvantage [47]. The scheme was rapidly expanded with funding allocated to respond to challenges during the COVID-19 pandemic. Subsequently, 25% of primary and secondary schools whose students faced the greatest number of barriers to participating in education, determined by a Ministry of Education equity index measure, were eligible for the free lunches. The scheme was given the Māori name, Ka Ora, Ka Ako, which is explained as “in order to be in a good space to learn you need to be healthy and well” [48].

Ka Ora, Ka Ako was organised around multiple goals: social, nutritional, and educational, as well as encouraging environmental sustainability. The meals were universal within schools to avoid stigma, but there was diversity in how schools chose to source meals [48]. Meal providers were required to pay the living wage to staff. Many schools (but not all) accessed small-scale local providers, or chose to prepare their own meals, using the funding allocated. Some schools worked together and commissioned catering companies. The flexibility of arrangements was seen as a strength of the scheme, allowing schools to create meals that worked well for their own communities [48]. In 2023, approximately 27% of school children were receiving a meal [49].

Evaluations of the scheme show positive outcomes in relation to ameliorating hunger, improving mental and physical health, and reporting improved behaviour [47,50]. Limited data suggest improved attendance, although this is complicated by the impacts of the COVID-19 pandemic [51]. Some researchers highlighted that the scheme only reached children attending schools that had high numbers of children living in lower socio-economic homes, despite a proportion of children experiencing food insecurity attending schools in higher income areas [52] (p. 10). This situation highlights how a universal scheme is the only full insurance against some children in need missing out. At any rate, Ka Ora, Ka Ako provided a model that could have been expanded to all schools over time.

Reweti et al. 2025 [53] provide an example of how Ka Ora, Ka Ako supported multiple objectives within a school, including cultural, social, health and academic aspirations, uplifting the environment in the school beyond merely providing nutrition. The school could organise the food through practices and spaces that were grounded in Māori cultural practices, repurposing a building to serve as a ‘wharekai’ (a traditional space for sharing communal meals, grounded in core cultural values), which supported students to share responsibilities, and created a reciprocal social experience around the meals [53]. The school created a form of cultural reconnection, relational practices that reinforce Māori ways of being [54]. The researchers conclude that the lunch programme operates within the school as a “multi-dimensional health promotion setting” [53]. The introduction of Ka Ora, Ka Ako supported health and educational improvements but could also build capabilities and agency for students and integrate multiple positive outcomes over the longer term.

#### 1.1.5. Contested Policy: A New Approach to School Meals

In March 2024, the National Party-led coalition government announced that there would be a review of Ka Ora, Ka Ako. There was immediate concern about the future of the programme because the minister appointed to review the school lunches was David Seymour, Associate Education Minister and critic of the existence of the programme whilst in opposition [55]. For example, a coalition of public health experts told the media that: “We are concerned that we have a minister in charge who has made it clear he wants the programme shut down for ideological reasons without full consideration of the evidence. His appointment signals that the future of the programme is in question, which is extremely concerning because it would jeopardise the health and wellbeing of these kids.” [56]. Fears about the review were reinforced when the minister requested evidence of measurable outcomes from the scheme but prioritised attendance data rather than broader benefits, and signalled he planned on cutting funding to the programme by at least 50% [57].

Instead of cancellation or targeting, the minister announced staged changes, to begin in 2024, with the final stage in 2026, that would focus on cost savings and efficiencies but deliver to additional early childhood settings [58]. Cuts from over $300 million in spending to $170 million were to be achieved through a centralised model, whereby large-scale commercial suppliers deliver meals [58]. The meals were developed to meet the contract price through economies of scale, which is achieved through mass-produced meals and the use of contracting to lower wage costs [59].

The new scheme almost immediately encountered logistical issues relating to the delivery of meals nationwide, which left a proportion of schools receiving delayed or inconsistently heated meals [60]. In some cases, lunches arrived towards the end of the school day [61]. Additional problems emerged with the repetitive menu, inadequate portions, and unappetising nature of the meals, which resulted in high levels of waste in some schools [62]. The minister described such problems as ‘teething issues’, despite many of the difficulties still occurring many months into the changes [63]. More seriously, specific incidents emerged of spoiled or burnt food, damaged and leaking packaging, and pieces of plastic in meals [64]. In one school, a child suffered burns from a hot meal, and teachers reported that the packaging was difficult for children to safely manage when food was excessively hot [65]. It also emerged that the halal meals were not certified halal [66] and meals labelled vegan contained beef [67].

In light of the changes between the original Ka Ora, Ka Ako iteration of school lunches and the reformed scheme, this present study deployed narrative methods to examine how the problem of hungry children in schools, and responses to this problem, were conceptualised within media and policy. Rather than pre-determined hypotheses, the study drew on the ethnographic concept of ‘fore-shadowed problems’ [68], approaching the investigation of the phenomenon of school meals in Aotearoa New Zealand with some questions to guide an unfolding analysis without restricting the findings: ‘Why were state-funded lunches not introduced until 2020, despite food insecurity rising from the 1990s?’; ‘How were the issues of children going hungry being characterised?’; ’How might dominant understandings about the nature of the problem of children going hungry at school shape the responses or solutions to the problem?’; and ‘How do the stories about hungry children in schools connect with broader stories about poverty and food insecurity?’

## 2. Methodology

Community psychology researchers typically frame their work within a scholar-activist tradition, often conducting research within a social justice framework rather than aspiring to ‘neutrality’ [69]. The impacts of unequal power relations between groups in society are a focus for community psychology research, given the extent to which such unequal relations can result in material and psychosocial harms for marginalised groups. This present paper is based on analysis of how the nature of the problem of hungry children in Aotearoa New Zealand schools, and responses to that problem, were characterised within and across mainstream/legacy media and policy because such characterisations inform the range of possible solutions to the problem, yet tend not to include the voices of those living with poverty and precarity [70]. The recognition of historical dimensions to the research topic is also congruent with community psychology scholarship. Nelson and Prilleltensky (2010) argue that social problems in the present have “deep historical roots” (p. 26), indicating the importance of placing issues within their context [69]. History informed critical analysis, by highlighting the historical context for culturally dominant assumptions about an issue like hunger in schools.

In addition, the research was guided by an interpretive ethnographic orientation. In particular, the methods were inductive and iterative, casting a wide net in terms of collecting initial data, and remaining open to the emergence of notable features of the data during overlapping collection and analysis phases [68]. Ethnographers traditionally situate themselves within a bounded setting and treat data collection as an immersive and flexible process. In this case, the researcher was not located within a physical research site, but the collection of data followed an ethnographic model of actively weaving together knowledge from a diverse range of empirical materials [71]. This model recognises the researcher as a part of the research, producing knowledge, rather than describing an object of study [72]. In accordance with good practice in interpretive methodologies, I used reflexive fieldnotes to support critical engagement with my own research process. My positionality as a New Zealander of European descent required particular care with cultural concepts and worldviews, and I was grateful to be assisted in this by academics within the Māori Psychology Research Unit at my university.

### 2.1. Narrative Methods

Narrative methods were employed in order to identify how stories functioned across a diverse range of materials. Narrative methods are well-established across the social science disciplines, reflecting the foundational role of stories in how human beings create and share meaning [73]. Stories form a means for sharing understandings of the world and ourselves, through culturally comprehensible narrative forms. Narratives order events into a sequence and format that helps us make sense of phenomena, whether at the level of individual biography, or in terms of macro-level developments [74,75].

In this study, media, policy, public relations and advertising are all implicated in constructing stories about the scale of hunger within schools and feeding children in schools. Rappaport (2000) highlights how some stories become culturally authoritative, legitimised across different settings in media, policy, and institutions, attaining status as dominant narratives, which serve to marginalise alternative stories [75]. The stories that attain cultural dominance within a specific socio-historical setting consequently reflect power dynamics, leading Rappaport to work with communities to elevate alternative stories. In the case of the present study, the focus for investigation is the authoritative or dominant narratives that give shape to how social issues are responded to. Despite sometimes attaining ‘common sense’ status, it is worth stating that dominant narratives are resources for understanding social issues, not completely deterministic in how they act upon communities and individuals, who still have some agency to resist or mobilise narratives [75]. The focus on dominant narratives in this study does not denote an absence of any counter-narratives. However, the choice was made to foreground narratives most closely aligning with policy developments.

### 2.2. Data Collection

The data collection and analysis for the present study comprised two parts. Data for part one were drawn from a broader critical multi-level investigation into school milk that incorporated narrative analysis of how hungry school children and their families were being characterised prior to the 2019 introduction of state-funded lunches. Part one began with an examination of primary and secondary historical sources, investigating how poverty, hunger, and charity were conceptualised in Aotearoa New Zealand from 1850 to the 1930s, to provide context for how approaches to poverty developed post-colonisation by European settlers.

Subsequently, searches were conducted for New Zealand media, policy and corporate material produced between 2000 and 2018, using search terms: ‘hungry children in schools’; ‘hunger in schools’; ‘feeding children in schools’; ‘food in schools’; ‘milk in schools’; and ‘child poverty’. Initial data selection occurred when I filtered out items that did not fit the topic of the project (for example, items about hungry children referring to lunchbox recipes for busy parents). Further items were identified through the evolving insights of the study, rather than predetermined sampling criteria. In addition to text-based media and policy, I collected broadcast items during the period 2014–2018, and political speeches given in face-to-face meetings by the four main political parties, during election campaigning in 2014.

The resulting corpus for analysis comprised the following: (1) 103 text-based items from mainstream/legacy national and regional news; (2) 26 items from broadcast radio, television news, and current affairs programmes; (3) 36 policy items, for example reports, briefings, working groups and reviews, political speeches, and parliamentary debates; and (4) 30 items produced by charities, corporates, social enterprises, and partnership schemes with a focus on addressing children’s hunger. In keeping with a narrative orientation, visual elements within the data (such as imagery, photographs, and illustrations) were considered part of the storytelling within the materials. Narratives can be communicated with visual elements, or form part of how a story communicates meaning and function [76].

Part two of the data collection and analysis occurred after the introduction of state-funded meals in schools, with a particular focus on media coverage relating to changes to the school lunch programme from 2024 to 2026. The search terms for part two were for items from March 2024 (when changes to the scheme were signalled) until March 2026: ‘lunches in schools’; ‘Ka Ora, Ka Ako’; ‘changes to school lunches’; ‘food in schools’; and ‘new school lunch programme’. The resulting 30 items of policy, press releases, and text and broadcast media relating to changes to the scheme provided material to bring into conversation with the earlier research findings in part one. The intent of bringing together the two parts of this study was to investigate how earlier narratives about hunger in schools and how to address the issue, which had preceded the introduction of Ka Ora, Ka Ako, were discernible or distinct from the evolving stories about changes to food in schools from 2024 to 2026.

### 2.3. Analysis

The analysis for part one was iterative, identifying dominant stories within the data first, rather than applying pre-coded themes. Narrative analysis is intentionally flexible because stories are context-dependent constructions of meaning [76]. Instead of applying a fixed sequence of steps or coding, the researcher uses interpretive readings to understand how meaning is constructed through the form and function of stories, avoiding fragmentation of the narratives. Narratives emerged through repeated readings, grouping stories together, and creating mind maps of key features within the data. The focus was to identify where stories appeared across different types of empirical data, indicating how some ways of storying relevant issues were resonating most prominently. The immersive process of analysis eventually led to further insights into the nuances of how interconnected narratives also contained distinctive elements. Eventually, stages of grouping and mapping narratives across empirical materials illuminated overarching narrative tropes prior to the introduction of Ka Ora, Ka Ako.

Part two applied narrative analysis to the material from 2024 to 2026. However, part two was additionally guided by the findings from part one. The focus for part two was to understand how the previous findings informed the newer developments around school meals. This analysis was facilitated by mapping key elements of stories from part one and part two together, and examining inflexions or more substantial shifts in how stories were taking shape during the process of reforms to the lunch programme. Overall, the goal was to develop a rich, contextualised understanding of the stories occurring across the empirical materials [68] (pp. 239–262).

## 3. Results

Analysis identified dominant narratives shaping understandings about the issue of children going hungry in schools and which solutions might be appropriate. Interconnected stories that resonated within and across the different sets of research materials became recognisable in terms of their characterisation of the problem, its predominant causes, and actions or policies advocated on the basis of these diagnoses of the issue. Prior to the introduction of Ka Ora, Ka Ako, even the existence of poverty and food insecurity in Aotearoa New Zealand was a disputed concept.

### 3.1. Denying or Minimising the Existence or Scale of the Problem of Hunger in Schools

The existence of poverty in Aotearoa New Zealand is contested within stories that emphasise how the nation is an assumed ‘land of plenty’, where ‘true’ poverty is not found. Concepts of absolute, versus relative poverty contribute to these understandings. Absolute poverty is generally defined as a type of poverty often seen in lower income countries where populations are deprived of clean water, shelter and other basic necessities, resulting in immediate threats to health and life [77]. Relative poverty refers to the standard of living within a country and to people who fall below this expected level in relation to how others are living [77] (p. 23).

In terms of how the issue of school children going hungry is being storied in relation to poverty and food insecurity, some commentators build arguments based on the assumption that ‘relative’ poverty is not poverty at all. This can take the form of comparing the suffering of those living at subsistence level globally with the assumed less arduous experiences of poverty locally. Relative poverty also functions to minimise claims of hardship by suggesting that the application of poverty measures is constructed statistically to place anyone below a certain median income figure into the category of ‘poverty’ [78]. The issues of food insecurity, poverty, and hunger in schools become debates about measuring, quantifying, and evaluating whether these issues are valid problems.

Political framing of the issues includes obfuscating the data on growing levels of poverty and food insecurity, especially by governing parties during the period when child poverty was rising sharply [77]. One method was shifting the focus from evidence to anecdote. For example, in 2014, then newly re-elected Prime Minister John Key stated that government funding for school meals is not necessary because: “the feedback we get around lunch is that the vast bulk of children do come to school with lunch and while there are one or two that don’t, the schools understand that and they deal with that situation very discreetly” [79]. The Prime Minister is minimising the nature of the problem and suggesting that schools are capable of managing it.

### 3.2. Blaming Parents

Parents and parenting form a key focus for dominant understandings about the issue of children going hungry in schools. Many media stories sharing the concerns of teachers and school principals about the issue within schools produce comment sections where readers express their conviction that parents are to blame. The Minister for Social Development, Paula Bennett, listed the reasons why children were hungry: “… problem debt, substance abuse, poor decision-making, and a history of neglect were common reasons why some parents were not taking full responsibility for their children” [80]. The trope of a wilfully negligent parent appears, as do characterisations of parents as ignorant, uneducated and unskilled [81]. The irresponsible parent is deemed uncaring and an object of moral censure, while the incapable parent is portrayed as being in need of education to amend the deficits in their budgeting, shopping, cooking, or gardening skills [82]. The notion that providing food to children in schools merely perpetuates the problem of parental neglect by alleviating parents’ responsibilities provides a logic for not supporting school meals [83].

### 3.3. Welfare Dependency

Locating issues of hungry children in schools at the level of dysfunctional individual parents and families connects with the concurrent idea of ‘welfare dependency’ [84]. This narrative decries how paying social security benefits to people who are unemployed, caring for others, unwell, or disabled has created an intergenerational deficit. The argument is often that the ease with which people can attain money without being in paid work is fundamentally damaging the fabric of society and undermining individual capability and responsibility [85]. Parents could acquire paid work to remedy child hunger. The combination of blaming individual parents and the assumption that poverty only occurs within households supported through state benefits maintains the idea that opportunities are available if parents ‘help themselves’. Single mothers are a particular target for judgement [86]. There is also a racialised element to some of the ways parents accessing welfare are characterised. A notable example in debates preceding the 2012 ‘Feed the Kids Bill’ was the publication of multiple racist cartoons in a mainstream newspaper depicting Māori and Pacific parents rejoicing in their ability to remain indolent while the state feeds their children [87].

### 3.4. Taxpayers Are Not Responsible

Arguments for the introduction of a state-funded school meal programme in Aotearoa New Zealand are often countered by depicting such a programme as unfair on taxpayers, who are required to fund school meals. Taxpayers are characterised as hard-working citizens, beleaguered by excessive requirements to pay for alleviating problems not of their own making, for example, providing food for other people’s children [88]. Personal philanthropic choices to donate time or money to charity schemes are sometimes framed as preferable to state-funded solutions [89]. The role of the corporate sector is also proffered, as sponsors, benefactors or partners with the non-profit or government sector businesses can promote products and fulfil ‘corporate responsibility’ remits, while saving taxpayers from fully funding school meals [90]. There is also a connected assertion that communities have become dependent on state initiatives and have consequently lost capability to find their own solutions—for example, the following comments on the Maxim website, a pro-business organisation: “So what happens when you replace community initiative with government handouts? … It should be communities at the heart, not schools. And definitely not the state.” [91].

### 3.5. Heroes Feeding Children

Some stories focus on the good work of compassionate people who are helping to alleviate hunger within schools [92]. ‘Heroes’ are busy with the practical work of feeding children at school, in the absence of a state-funded meal programme. Media reporting about individuals, corporations, or charities responding to children’s needs were amplified by politicians seeking to demonstrate that ‘something was being done’ in response to the issue. A Member of Parliament commented in a parliamentary debate about whether to fund food in schools, that ‘We have some great examples of organisations stepping in to do their bit to support families. We applaud companies…’ before naming and praising the brand names concerned [93]. There were a significant number of such ‘heroes’ rescuing school children from hunger, prior to the introduction of Ka Ora, Ka Ako, some of whom continued to engage with schools to provide additional food, such as breakfasts or snacks.

### 3.6. From Ad Hoc to State-Funded Meals: Pragmatism and Rights to Education

It is important to recognise that alongside the more individualised approaches evident above, there are other understandings of how to define and respond to hunger in schools that gained traction and eventually supported the development of a state-funded scheme by the Labour (Left-leaning) coalition government elected at the end of 2017. Critically, pragmatic arguments for a state-led response became more tenable once the scale and nature of the problem became harder to ignore, following sustained media attention and growing public disquiet. Sidestepping debates about who or what is to blame, the focus shifts to the impacts on children within a school, including behavioural and learning consequences when children are not fed. The following comment from then Labour Party leader David Shearer indicates this standpoint: “I hear people argue that this is the responsibility of parents. We can debate that endlessly but it won’t change this reality: tomorrow morning kids will still turn up to school hungry. And a hungry kid is a distracted kid who can disrupt an entire classroom” [94]. Staff within schools keenly emphasised the practical issues they were facing on the ground, particularly in schools with the greatest levels of poverty in their communities [95]. Future costs of not ensuring children can attend and achieve at school are also part of this story [96].

The emphasis on classroom pressures also connected with broader concepts of children’s rights to equitable access to learning. Children’s rights to participate at school are supported by adequate nutrition, but there is also a recognition that children are never responsible for their own poverty. A child-centred approach to policy draws from ideals of fairness and investment in the future generation, which creates the possibility for school meals as one means for addressing impacts of child poverty [41]. The focus on children is key, given their blameless position within the context of how parents and communities are being characterised within dominant narratives.

### 3.7. 2024–2026 Reforms

The stories explored above continue to shape understandings and policy when the original school meal scheme is reviewed and subsequently reformed by a National-led (right leaning) coalition government, elected in 2023. Some elements receive stronger emphasis when changes to the school meals are foreshadowed or promoted.

#### 3.7.1. Reasserting Parental Responsibility

Parental responsibility is emphasised, despite the announcement that school meals will continue to be provided in the same number of schools as previously. In addition, the idea that the meals are not required as a result of poverty, but rather due to parental irresponsibility is evident, for example, in comments by the Prime Minister, Christopher Luxon, when asked about ongoing quality issues with the new meals: “If you really are unhappy with it, for God’s sake, go make a Marmite sandwich and put an apple in a bag just like you and I had.” [97]. The suggestion is that parents should not complain about low-quality school meals and could instead send a sandwich, indicating that he thinks parents could provide food if they chose to.

#### 3.7.2. Minimising Costs for the Taxpayer

Emphasis on maintaining targeted approaches to alleviating food insecurity and a significant reduction in per-head funding for school meals are framed as prudent management of resources for taxpayers [58]. The new meals remove previous goals around paying a living wage to external suppliers and favouring environmentally sustainable options; instead, the lowest price for delivery of the scheme is the key criterion. The focus on minimising costs defines Ka Ora, Ka Ako’s broader social goals as luxuries no longer affordable.

#### 3.7.3. Settler Culture Reinvigorated

The announcement and press about the plans for new meals characterised foods purportedly offered in Ka Ora, Ka Ako meals as ‘woke’, or needlessly ‘exotic’. The minister, David Seymour, announced the changes to the programme on social media by stating that the programme would do more with less money by “feeding kids the fruit and sandwiches their parents would, not woke food like quinoa and sushi” [98]. The framing of perceived extravagance within the Ka Ora, Ka Ako meals is achieved through building stories about the contrast between the foods that ministers in government ate for their own packed lunches during the 1970s and 1980s (basic sandwiches and a piece of fruit, seemingly) with more recently popular foods in Aotearoa, such as sushi or humous [98]. The entertaining nature of these contrasts appealed to the media, but they are stories that serve to re-establish a nostalgic dominance of predominantly British-origin foods and create the impression that children had been dining on ‘exotic’ treats at the expense of the taxpayer [99]. The ‘back to basics’ story implies that the group of children who are seeking free lunches have been expecting decadent foods that the hardy individuals of yesteryear did not require.

In a symbolic development, in 2025, the Māori name, Ka Ora, Ka Ako, which expressed aspirations for children’s wellbeing, has been supplanted by the English name, ‘Healthy School Lunches’ [100]. The change is presented as part of the ‘back to basics focus’ but also reflects a broader political trend to reverse the integration of more Māori language into everyday usage.

#### 3.7.4. Resistance: In Defence of Ka Ora, Ka Ako

In the midst of the review of school meals and the signals coming from the minister in charge of the review, stories about the values, connections, communities and nutritional benefits of Ka Ora, Ka Ako emerged and were elevated within the media [56,101]. A coalition of public health, medical, and academic groups produced evidence for the benefits of continuing the school meal scheme [102]. The narratives in support of Ka Ora, Ka Ako emphasised its role in supporting child health, wellbeing and learning, situating the scheme as a public health initiative with long term benefits [51]. Public health narratives offered resistance to the political rhetoric around minimising costs in the present as the key goal and built a case around the value of nourishing children well to avoid future costs.

## 4. Discussion

This study demonstrates how dominant understandings about the causes of the growing number of hungry children in schools, and how to respond to hunger in schools, are shaped by history. The findings, although situated within the context of a school meal programme in Aotearoa New Zealand, highlight how local policy development takes place within broader stories that emerge within neoliberal ideology globally. Narrative tropes that build and sustain neoliberal definitions of social problems and their origins provide the foundations for locally inflected versions of individualised and market-based ‘solutions’ [31]. The adaptive nature of many neoliberal tropes is one of the reasons that characterisations of groups that are blamed for their poverty are durable and can be deployed in different contexts [103].

### 4.1. Responsibilisation

One of the key findings was the tendency to blame individual parents for not providing food to their children, despite the evidence for increasing numbers of families being unable to afford basic costs, such as rent, bills, medical appointments, transport and nutritious food for all household members [77]. Historically, irresponsible parents have been the subject of moral censure. Nonetheless, responsibility has become an important signifier of neoliberal reconfigurations of the relationship between citizens and the state in countries like Aotearoa New Zealand, where the welfare state is under neoliberal revision [104]. Responsibilisation refers to a process by which individual responsibility is elevated as a means to diminish state or collective responsibility, signifying a shift toward individual freedom of choice [105]. In the context of Aotearoa New Zealand’s settler colonial culture, the ideal of a self-reliant individual is promoted, which reinforces the responsibilisation of parents within dominant stories, instead of more relational and collective approaches to sharing responsibilities.

Blaming parents locates the issue at the level of individual parents or families, rather than recognising upstream, structural drivers for food insecurity, or how food insecurity impacts some groups more than others. The effect is to ‘other’ the parents of children who are attending school without food, constructing such families as fundamentally deficient in comparison to more affluent families, in terms of their values and behaviours [106]. The findings show how this ‘othering’ can have classed, gendered and racialised dimensions, for example, in the racist cartoons published in Aotearoa New Zealand, and in commentary about single mothers draining taxpayers’ money. Negative stereotypes of parents living in poverty, and characterising parenting deficits as the drivers of children’s food insecurity, are evident in many neoliberal societies [107,108].

### 4.2. Welfare Dependency

The findings show how a focus on parents and their choices are enmeshed within neoliberal rhetoric that characterises ‘dependency’ as an illegitimate state, particularly ‘welfare dependency’. Welfare dependency functions within broader neoliberal ideological narratives about poverty. Neoliberal governments have promoted paid work as a moral and economic imperative, cutting funding for social security and tightening conditionality around state benefits [109]. Despite many children experiencing poverty living in homes where one or more adults are in paid work, or where paid work is supplemented through the benefit system due to low wages, ‘welfare dependency’ is often presented as the cause of poverty [110]. The idea of intergenerational worklessness connects with the aforementioned deficit framing of parents and their communities. The denigration of ‘dependency’ also resonates with the settler colonial assumption of plentiful opportunities for those who work hard and achieve desired independence. The interdependence of all human beings is forgotten within this narrative, as is the invisible, unpaid domestic and care labour upon which paid workers depend [111].

### 4.3. The Deserving and Undeserving

Children are framed as deserving of compassion in Aotearoa New Zealand, as in most other places. However, the findings show that compassion can be reserved for children, while adults who are living with poverty are held responsible not only for their own hardship, but for inflicting hardship on their children. Even the notion of ‘child poverty’ that is mobilised in support of addressing children’s suffering reinforces a distinction between the deserving child and others in society [112]. The model of separating children’s needs from those of their families does not align with Māori values, which integrate children within collective responsibilities and emphasise relational ways of being [30].

### 4.4. ‘Austerity Neoliberalism’ and Weaponised Nostalgia

From 2024 to 2026, narrowing the goals of the school meal programme and engaging large multinational providers has reduced the cost per meal, but with reduced quality. The concept of ‘neoliberal austerity’ highlights how the state contracts expenditure on public services and civic spaces in the name of managing economic crises [113]. Austerity is applied in service of neoliberal goals that promote privatised and market-based solutions, rather than state expenditure to support the public good [114]. Minimising costs to ‘the taxpayer’ has become the guiding principle for the new lunches’, during a period of deepening food insecurity [115]. Austerity logic is deployed to keep the school meals at a level of bare sustenance.

In addition, the necessity of taking the meals ‘back to basics’ was promoted by referencing foods that were eaten in the past in Aotearoa New Zealand schools. Sandwiches from home were reanimated as appropriate foods, in contrast to perceived ‘woke’ foods adopted from other cultural settings [98]. The interesting aspect of these juxtapositions was that the meals eventually delivered to primary schools in the new scheme were pre-heated meals, including butter chicken, chicken katsu, and bolognaise. The choice of hot meals was made on the basis of what the catering companies tendered within the price range, which turned out not to be the simple sandwiches that seemed to be signalled initially by politicians. The ‘back to basics’ rhetoric was not connected with the food itself; rather, it was a story about how parents in the past could provide basic lunches and that was a better time, when families were self-reliant. The message is about how the state in the present period should not really have to provide meals in the first place. The framing is redolent of a charity model, a crisis response for which recipients should be grateful, rather than the state ensuring that all children can thrive [31].

### 4.5. Community Psychology and Food in Schools

A critical community psychology lens highlights how neoliberal and settler colonial tropes are shaping school meal reforms. More importantly, a community psychology approach moves the conversation about school meals away from deficit framing and short-term cost cutting, animating the possibilities for treating access to decent nutrition as a human right and schools as sites for all children to flourish. A stronger grounding in social justice and Māori rights to self-determination, along with secure funding, could have provided Ka Ora, Ka Ako with more stable foundations. Nevertheless, it is important to recognise that state-funded school lunches are in place.

A community psychology approach to school meals is exemplified in the example of a school that drew on Ka Ora, Ka Ako resources to create a school meal grounded in cultural practices, social connection, and health promotion [53]. The example demonstrates how settings such as schools form important adaptive systems within health promotion, something also embedded within the Finnish model of school food [17]. In 2021, a broad-based global coalition, co-led by Finland and France, was formed to support a public health evidenced strategy in school meal provision globally. The goal of every child receiving a healthy and nutritious meal at school by 2030 has been set, signalling a shift towards more universal school meal provision as a global public health measure [116]. The coalition positions collaboration and collective action as crucial factors in supporting countries to provide the health and equity improvements that countries like Finland have demonstrated.

### 4.6. Limitations and Future Research

Inevitably, a qualitative study must foreground some stories more than others. This study focused on dominant narratives and less on resistance or stories of lived experiences. The presence of counter-narratives is acknowledged, but they were afforded less space in order to ensure that the key findings were given sufficient depth of analysis. Likewise, it was not possible to incorporate all of the nuance in relation to ways in which Aotearoa New Zealand society developed within the conditions of colonisation, including some periods when egalitarian ideals were more evident.

In terms of methods and materials, the data were drawn predominantly from mainstream/legacy media, formats that are less relevant for future research. Future studies could focus on the more dynamic and interactive landscape of social media, especially given its growing popularity as a source of news and commentary. Building understanding of how the new media environment interacts with people’s pre-existing assumptions would provide further insights.

## 5. Conclusions

This study has applied a critical community psychology lens to dominant understandings about responding to hungry children in schools. The findings highlight how the introduction of a state-funded school lunch scheme reflects its historical context. Settler colonialism and neoliberalism are implicated in the structural drivers of poverty and food insecurity and also the micro-level distribution of meals to school children. The creation of Ka Ora, Ka Ako was able to move Aotearoa New Zealand beyond ad hoc responses to hunger in schools, towards recognition of collective responsibility for addressing the impacts of food insecurity. Nonetheless, the introduction of Ka Ora, Ka Ako took place within the context of dominant narratives that undermined commitment to embedding and institutionalising an optimal scheme. The introduction of Ka Ora, Ka Ako consequently had little time to become established, before deepening austerity neoliberal political narratives justified reforms that undermined the original aspirations for Ka Ora, Ka Ako. Consequently, the burnt and insufficient meals many children currently endure in schools are a grudging crisis response, embodying little care for recipients. In terms of community health, reinvigorating stories about equity and fairness for all children, from which Ka Ora, Ka Ako drew some support, could begin to rebuild a more inclusive school meal programme that supports all children to flourish. The more substantial steps will be shifting focus upstream, towards the historical inequitable distribution of resources between groups, and to advocate for Māori to regain control over their own food security.

## Data Availability

The original contributions presented in this study are included in the article. Further inquiries can be directed to the corresponding author.
